# A Novel Image Processing Method for Obtaining an Accurate Three-Dimensional Profile of Red Blood Cells in Digital Holographic Microscopy

**DOI:** 10.3390/biomimetics8080563

**Published:** 2023-11-22

**Authors:** Hyun-Woo Kim, Myungjin Cho, Min-Chul Lee

**Affiliations:** 1 Department of Computer Science and Networks, Kyushu Institute of Technology, 680-4 Kawazu, Iizuka-shi, Fukuoka 820-8502, Japan; kim@ois3d.cse.kyutech.ac.jp; 2 School of ICT, Robotics, and Mechanical Engineering, Hankyong National University, Institute of Information and Telecommunication Convergence, 327 Chungang-ro, Anseong 17579, Kyonggi-do, Republic of Korea

**Keywords:** digital holographic microscopy, disease diagnosis, image processing, noise filtering, computational visualization

## Abstract

Recently, research on disease diagnosis using red blood cells (RBCs) has been active due to the advantage that it is possible to diagnose many diseases with a drop of blood in a short time. Representatively, there are disease diagnosis technologies that utilize deep learning techniques and digital holographic microscope (DHM) techniques. However, three-dimensional (3D) profile obtained by DHM has a problem of random noise caused by the overlapping DC spectrum and sideband in the Fourier domain, which has the probability of misjudging diseases in deep learning technology. To reduce random noise and obtain a more accurate 3D profile, in this paper, we propose a novel image processing method which randomly selects the center of the high-frequency sideband (RaCoHS) in the Fourier domain. This proposed algorithm has the advantage of filtering while using only recorded hologram information to maintain high-frequency information. We compared and analyzed the conventional filtering method and the general image processing method to verify the effectiveness of the proposed method. In addition, the proposed image processing algorithm can be applied to all digital holography technologies including DHM, and in particular, it is expected to have a great effect on the accuracy of disease diagnosis technologies using DHM.

## 1. Introduction

Recently, research on disease diagnosis using deep learning techniques has been actively studied by many researchers [1,2,3,4,5,6,7,8,9,10,11,12,13]. In particular, in the context of the pandemic caused by COVID-19 that occurred in 2019, several studies are being proposed for rapid diagnosis of this disease [4,5,6,7,8,9,10]. Among these studies, a study that analyzes the statistical data of three-dimensional (3D) profile of the tester’s red blood cells (RBCs) using deep learning technology for quick and simple disease diagnosis has also been proposed [8,9,10]. In these researches, 3D profile can be obtained using digital holographic microscopy (DHM) technology. Conventional microscopy can obtain 2D information of the object, but DHM can obtain 3D information of the object. In disease diagnosis using deep learning technology, a higher degree of freedom guarantees higher accuracy. Therefore, DHM has been widely used in field of the disease diagnosis using living cells. Recently, disease diagnosis using DHM and deep learning has been used to diagnose not only COVID-19 but also many diseases such as cancer [11], sickle cell disease [12], and malaria [13]. In particular, the technology proposed in [10] showed diagnostic accuracy for COVID-19, with a sensitivity of 90.00% and a specificity of 93.86%, similar to that of a rapid antigen test. Sensitivity refers to the probability of judging a positive as positive, and specificity refers to the probability of judging a negative as negative. This diagnostic accuracy is expected to increase as more data is learned, and if this technology had been used previously, simpler and faster diagnosis would have been possible.

DHM uses holography that records and reproduces 3D information of an object using the coherence of light [8,9,10,11,12,13,14,15,16,17,18,19,20,21]. Holography was proposed by Dennis Gabor in 1948 [22]. Since digital holography, a method of recording holography using an image sensor, was proposed by Joseph W. Goodman in 1967 [23], the development of technology has been accelerated. The digital holography technologies are being applied to many research fields, including 3D image visualization [24,25], image encryption [26,27], object recognition [28,29], interferometry [30], surface shape measurement [31] and digital holographic microscopy (DHM). DHM is widely used not only in disease diagnosis using cell analysis [8,9,10,11,12,13,14,15,16,17,18] but also in application fields such as microstructure analysis [19], microbiological research [20] and refractive index measurement [21] due to its advantage of being able to obtain 3D information from microscopic objects. In the field of disease diagnosis using cells, the accuracy of 3D information of cells is particularly important. Among these diseases, sickle cell disease or malaria cause significant changes in the shape of RBCs, so that the accuracy of disease diagnosis is not related to the detailed information of 3D profile [12,13]. On the other hand, in the case of a disease such as COVID-19, there is no significant difference between 3D profiles of RBCs of positive and negative testers. Thus, it is difficult to distinguish them visually, and accurate data of 3D profile is essential when diagnosing with statistical data [8,9,10]. This is because minute errors in cell height information can cause errors in the diagnosis of diseases. Therefore, in disease diagnosis technology using RBCs, detailed and accurate height information is essential as the presence or absence of disease is determined by the difference in minute volume information of RBCs. In addition, if disease diagnosis research using detailed 3D profiles of RBCs develops, there is a possibility that deep learning technology can distinguish more diseases. To obtain accurate 3D information of RBCs, it is necessary to maximize the high-frequency information by widening the area of the windowed sidebands in the Fourier domain. However, in the Fourier domain, not only the sideband but also the direct current (DC) spectrum is widely distributed, resulting in noise of 3D profile. To prevent such noise, in the current disease diagnosis research using DHM, approximate RBC height information obtained from a narrow sideband is used [8,9,10]. Alternatively, a technique of using 3D information of the RBC by windowing sidebands as wide as possible and filtering noise have also been used [15,17]. However, this method also has a disadvantage in that not only noise but also high-frequency information of an object can be crushed. Therefore, it may not be said to be accurate height information of an object.

To solve this trade-off and improve the accuracy of disease diagnosis, in this paper, we propose a novel DHM image processing method for randomly selecting the center of the high-frequency sideband (RaCoHS) in the Fourier domain. Our proposed method sets the maximum area that can be windowed in the Fourier domain and uses multiple windowed sidebands centered on a number of randomly selected pixels within a region. Using these multiple windowed sidebands, we can calibrate the height information of the RBC. This algorithm has the advantage of using only windowed sidebands containing high frequencies obtained from the Fourier domain, and filtering noise while retaining high-frequency information. In addition, the RaCoHS algorithm has the advantage that it can be applied to not only RBCs but also the other micro-objects. In this paper, RBCs are used to compare 3D profiles acquired by existing methods and 3D profiles acquired by proposed methods, respectively, to confirm the filtering effect. Then, it is verified through numerical comparison using 3D profile of the microsphere by DHM with 3D profile of the ideal microsphere.

This paper is organized as follows. In Section 2, we describe the principles of DHM, general image processing methods, and our proposed method. Then, the experimental setup is described in Section 3. In Section 4 and Section 5, we show the experimental results and conclusions.

## 2. Theory

### 2.1. Digital Holographic Microscopy (DHM)

As we mentioned in Section 1, digital holography uses the coherence of light. This is because human eyes and the conventional image sensor cannot recognize the phase information of the light. To solve this problem, digital holography, a method of superimposing two lights on one imaging plane to create an interference pattern, is used and phase information of light can be obtained using this interference pattern.

**Figure 1:**
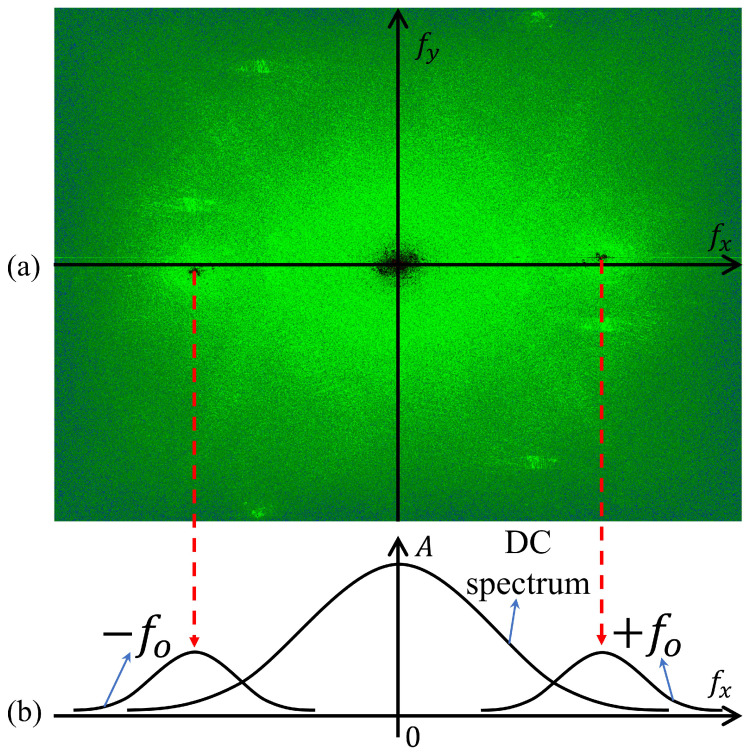
Fourier domain of the recorded hologram. (**a**) 2D and (**b**) 1D Fourier spectrums.

This interference pattern can be divided into a reference beam serving as a reference and an object beam having phase information scattered by an object. The recorded image which has the superimposed state of these two beams on the image sensor is called the hologram $I_{Holo}$, and this can be expressed as follows [15,16,17,18,19]:

(1) $$\begin{array}{c} I_{Holo}={\left|R\right|}^{2}+{\left|O\right|}^{2}+R^{*}O+{\mathrm{RO}}^{*} \end{array}$$

where R and O are the complex amplitudes of the reference and the object beam, and $A^{*}$ means a complex conjugate of A, respectively. In the Fourier domain, ${\left|R\right|}^{2}+{\left|O\right|}^{2}$ is the DC spectrum and the $R^{*}O+{\mathrm{RO}}^{*}$ represents the positive and negative sidebands. In addition, Equation (1) can be derived as the following [15,16,17,18,19]:

(2) $$\begin{array}{c} I_{Holo}={\left|R\right|}^{2}+{\left|O\right|}^{2}+R^{*}Oe^{j\phi }+RO^{*}e^{-j\phi } \end{array}$$

where ϕ is a phase information of the recorded hologram. Besides, it can be recognized that the components of Equation (2) show the distribution in the Fourier domain of the recorded hologram as shown in Figure 1.

Figure 1a,b show 2D Fourier spectrum and 1D Fourier spectrum, respectively. In Figure 1b, $+f_{o}$ and $-f_{o}$ mean $R^{*}Oe^{j\phi }$ and $RO^{*}e^{-j\phi }$ of Equation (2), respectively. As shown in Figure 1, each sideband is symmetrically distributed relative to zero. Therefore, the phase information of the recorded hologram can be obtained from one sideband in the Fourier domain. In addition, when the recorded hologram has object information, it is called an object hologram. In contrast, when there is no object information, it is called a reference hologram. To obtain phase information from a recorded hologram, the real part and imaginary parts of the image must be distinguished. Equation (2) can be derived as follows by dividing the recorded hologram into the real part and the imaginary part [19]:

(3) $$\begin{array}{c} \begin{array}{c} I_{Holo}\left(x,y\right)=Re{\left[\Psi \left(f_{x},f_{y}\right)\right]}^{2}+Im{\left[\Psi \left(f_{x},f_{y}\right)\right]}^{2} \end{array} \end{array}$$

where, $\Psi (f_{x},f_{y})$ is the Fourier transform result of $I_{Holo}$. The quantitative phase information can be obtained by calculating the argument as follows [19]:

(4) $$\begin{array}{c} \begin{array}{c} \phi \left(f_{x},f_{y}\right)=arctan\left[\frac{Im[\Psi \left(f_{x},f_{y}\right)]}{Re[\Psi \left(f_{x},f_{y}\right)]}\right] \end{array} \end{array}$$

When acquiring an object image, the wavefront changes due to scattering due to the difference in refractive indices between the object and the surrounding medium, resulting in a change in the interference pattern. For this reason, a difference occurs in the phase of the reference image and the object image, and this is called phase difference. Phase difference information can be calculated using the phase information obtained from each hologram as [15,16,17,18,19]:

(5) $$\begin{array}{c} \Delta \phi =\phi_{O}-\phi_{R} \end{array}$$

where, $\phi_{O}$ and $\phi_{R}$ are phase information of the object and the reference holograms, respectively. Furthermore, the phase differences Δϕ can be expressed as [15,16,17,18,19]:

(6) $$\begin{array}{c} \Delta \phi =\frac{2\pi }{\lambda }\left(n_{o}-n_{S}\right)\times h, \end{array}$$

where, λ is the wavelength of the coherent light source, $n_{o}$ and $n_{s}$ are the constant refractive indices of the object and the surrounding medium, and *h* is the height of the object, respectively. Therefore, the height of the object can be obtained by rearranging Equation (6) as the following [15,16,17,18,19]:

(7) $$\begin{array}{c} h\left(x,y\right)=\frac{\Delta \phi (x,y)}{K\times \Delta n}, \end{array}$$

where, *K* is the wavenumber, and Δn is the difference in the refractive index.

The cross-section of the interference pattern shows the same pattern as the sine function, and the Fourier transform solution of the sine function is two symmetrical delta functions, so the information of the fringe pattern can be separated in the Fourier domain. However, it is difficult to create an ideal fringe pattern due to camera noise, optical device noise, etc., and as a result, the information in the DC spectrum also remains in the same Fourier domain. The problem arises that the DC spectrum and sideband are placed in the same space and is difficult to separate fringe pattern information only. As shown in Figure 1, since the DC spectrum is distributed over a wide range in the Fourier domain, it is difficult to obtain the phase information of an object by distinguishing only the sidebands as we mentioned earlier. For this reason, DC spectrum information is included in the windowed sideband, and noise is generated in the extracted height information of the object. When windowing the sideband with a wide range, since noise generated by the DC spectrum increases, it difficult to obtain accurate height information. In addition, to avoid noise caused by unnecessary information in the DC spectrum, it is best to place the conjugate term as far away as possible. However, for the DC spectrum and conjugate term to be far enough apart in the Fourier domain of the recorded hologram, the interval between the interference patterns generated by the reference beam and object beam must be sufficiently narrow. The result of setting up the interval to window as wide a range as possible is shown in Figure 1a. If we set up the optical system to be further apart than Figure 1a, it is easy to avoid unnecessary information due to the DC spectrum, but the range that can be windowed becomes narrow. This can also be said to be one of the trade-off problems of DHM technology. Therefore, to solve this problem, our proposed method will be described in detail in the next Section 2.2.

### 2.2. A Method for Randomly Selecting the Center of the High-Frequency Sideband (RaCoHS) in the Fourier Domain

Before explaining the proposed image processing method in this paper, the maximum range for windowing sidebands in the Fourier domain is first set. In this paper, the direction of the interference pattern is set as horizontally as possible to the direction of the vertical axis of the image sensor to window the widest range of sidebands. In addition, the maximum windowing range of the sideband is set to half the distance from the DC spectrum to the center of the sideband peak.

**Figure 2:**
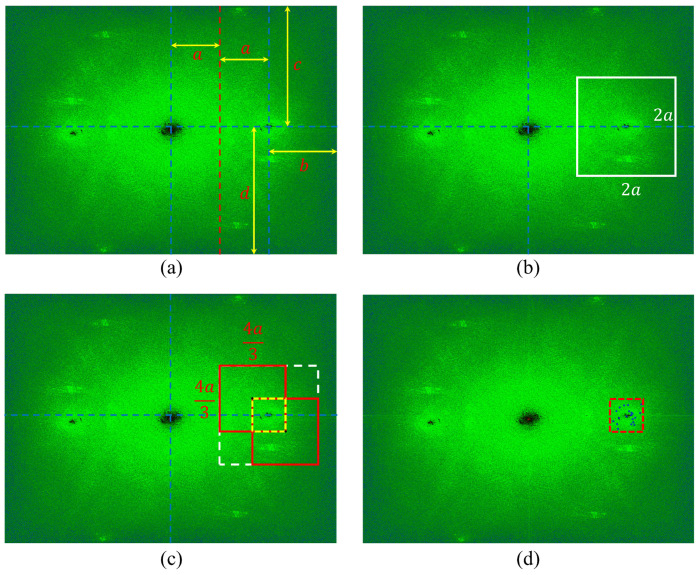
Method for randomly selecting the center of the high-frequency sideband in the Fourier domain. (**a**) Parameters for determining the maximum range of the windowed sideband, (**b**) the maximum range for windowing the sideband, (**c**) the maximum range for selecting the center of the windowed sidebands, and (**d**) the result of selecting 20 pixels randomly after setting the range of pixels.

Figure 2 illustrates our proposed method step by step. Figure 2a shows parameters for determining the maximum range of the windowed sideband. In Figure 2a, *a* and *b* are half of the horizontal distance between the sideband peak and 0, and the distance to the right resolution limit, respectively. *c* and *d* represent the distance from the peak of the sideband in the vertical direction to the upper and lower resolution limits in Figure 2a. Then, we select the minimum value among these parameters. Next, the twice value of this parameter is the length of one side, and the square centered on the sideband peak is set as the maximum range for windowing the sideband as shown in Figure 2b. In this paper, the size of the windowed sideband is set to 2/3 of the maximum range for windowing the sideband set in Figure 2b. However, the size of the windowed sideband can be defined differently depending on the user using this algorithm. First, one axis of the maximum range for windowing the sideband is defined as $R_{m}$, and one axis of the size of the windowed sideband is defined as $S_{W}$. Since the range of $R_{m}$ was determined based on the sideband peak, if the length of $S_{W}$ is less than $R_{m}/2$, there may be cases where the sideband peak is not included in the range of the sideband to be windowed. So, we can limit $S_{W}$ as follows.

(8) $$\begin{array}{c} \frac{R_{m}}{2}<S_{W}<R_{m}, \end{array}$$

As a result, no matter which pixel is selected in the yellow rectangle as shown in Figure 2c, the windowed sideband centered on that point exists within the maximum range for windowing the sideband. The closer the $S_{W}$ is to the $R_{m}/2$, the wider the range from which the center can be selected (yellow rectangle in Figure 2c), and the closer the $S_{W}$ is to the $R_{m}$, the narrower the range. Figure 2d shows the result of selecting 20 pixels randomly after setting the range of pixels that can be selected by our method. However, one of the selected random pixels is set to include the peak of the sideband. The number of randomly selected pixels $N_{R}$ can be set according to the need for the accuracy of the shape information of the object. Finally, accurate height information of the object can be obtained by averaging the height information from each selected windowed sideband. The equation of this method is as the following:

(9) $$\begin{array}{c} h_{RaCoHS}\left(x,y\right)=\frac{1}{N_{R}}\times \sum_{i=1}^{N_{R}}\frac{\Delta \phi_{i}\left(x,y\right)}{K\times \Delta n}, \end{array}$$

3D profile obtained by the proposed method has less noise than 3D profile obtained by general image processing method, and compared to the conventional filtering method, it has the advantage of being able to reduce noise while maintaining high-frequency information and can be filtered using only the information obtained from the recorded hologram image.

## 3. Experimental Setup

In this paper, we use the modified Mach-Zehnder interferometry using two spherical waves to make the interference pattern as narrow as possible. To verify the accuracy of the measurement of the DHM system, check the magnification using microspheres with a cross-sectional size similar to that of RBCs.

**Figure 3:**
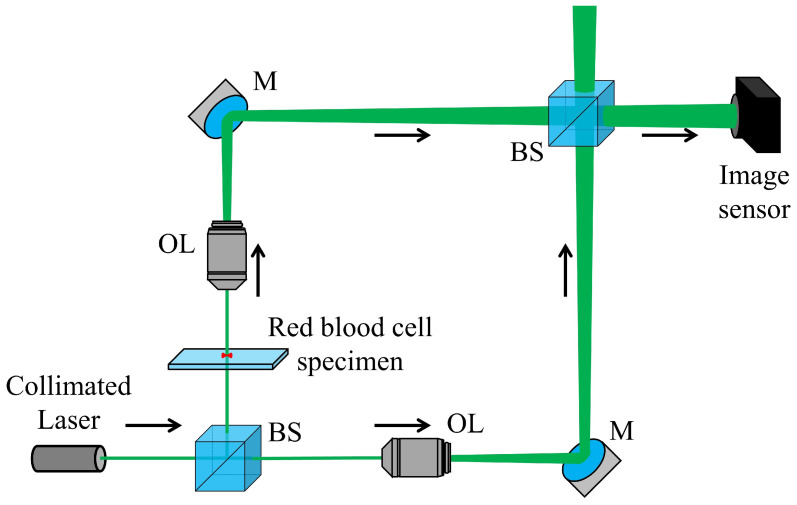
Experimental setup. M: mirror, BS: beam splitter, and OL: objective lens.

Figure 3 illustrates the experimental setup used to verify the effectiveness of the proposed algorithm. In this experiment, we used the collimated laser (CPS532, Thorlabs, Newton, NJ, USA) as the light source to reduce speckle noise, and the wavelength of this laser was 532 nm, and the output power was 4.5 mW. Both the object and reference beams were magnified by the 40× (0.65 NA) objective lenses. To record the hologram, we used a CMOS sensor (acA2500-14uc, Basler, Ahrensburg, Germany) with a pixel resolution of 2590 (H) × 1942 (V) and a pixel size of 2.2 μm (H) × 2.2 μm (V). We adjusted the interval of the interference pattern by rotating the beam splitter in front of the image sensor. We used RBCs from healthy males as a specimen. To prevent errors in height information caused by the overlapping of RBCs, a blood smear is made and used by spreading blood on a slide glass. After conducting the experiment using RBCs, we prepared and measured another specimen using uniform polystyrene microspheres with a size of 10 μm (02706-AB, SPI Supplies, West Chester, PA, USA) for numerical analysis. The certified mean diameter and the standard deviation are 10.04 μm and 0.66 μm from specification, respectively.

## 4. Experimental Results and Comparisons

**Figure 4:**
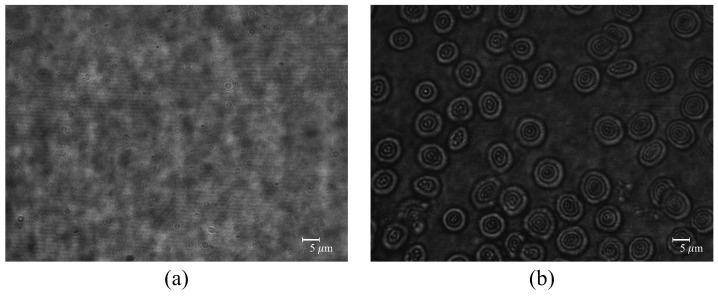
Recorded hologram images. (**a**) Reference and (**b**) Object images.

Figure 4 shows recorded hologram images. Figure 4a shows the reference image and Figure 4b shows the object image, respectively. We can obtain the height information of the RBCs from these hologram images. In this paper, we used a Goldstein phase-unwrapping algorithm [32] to obtain the phase information of the object. In addition, this experiment uses constant refractive indices of RBCs ($n_{o}$) and surrounding medium ($n_{s}$) with 1.42 and 1.34 (refractive index of the blood plasma in 532 nm wavelength), respectively [12].

**Figure 5:**
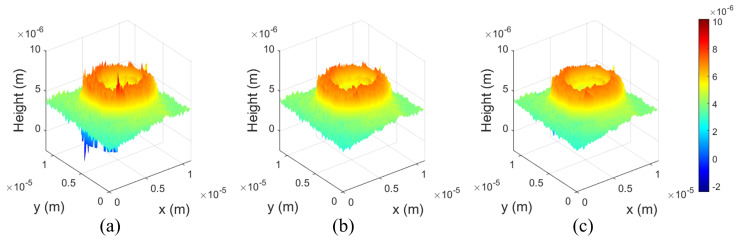
Reconstructed 3D profile obtained by (**a**) the conventional image processing method, (**b**) the proposed method ($N_{R}$ = 20) and (**c**) Gaussian filtering (σ = 2).

Figure 5 shows reconstructed 3D profiles by various methods. Figure 5a shows the 3D profile of the conventional image processing method for DHM, and Figure 5b shows the 3D profile of the proposed method. The number of the randomly selected pixels $N_{R}$ was 20. In addition, Figure 5c shows the Gaussian filtering result of Figure 5a. We can recognize that noise is generated randomly upper part and lower part of the object in Figure 5a. This random noise generated on 3D profile of the RBCs may give an error to a phase-unwrapping algorithm and may have wrong results in a technology that disease diagnosis using deep learning. On the other hand, 3D profile of the proposed method shows less noise than the conventional method as shown in Figure 5b. Moreover, 3D profile of the Gaussian filtering also shows better results than the conventional method as shown in Figure 5c. However, the principle of Gaussian filtering is a type of low-pass filtering such as averaging filter or median filter, and uses a filtering algorithm that reduces or removes the amplitude of high-frequency information. For this reason, the conventional filtering algorithms that are similar to Gaussian filtering may not be a solution to this problem. In contrast, the filtering algorithm proposed in this paper removes unnecessary frequency information from the DC spectrum without reducing or removing high-frequency amplitudes because it uses windowed sidebands of the same size windowed from different locations. Therefore, the proposed method can be said to filter while maintaining high-frequency information.

**Figure 6:**
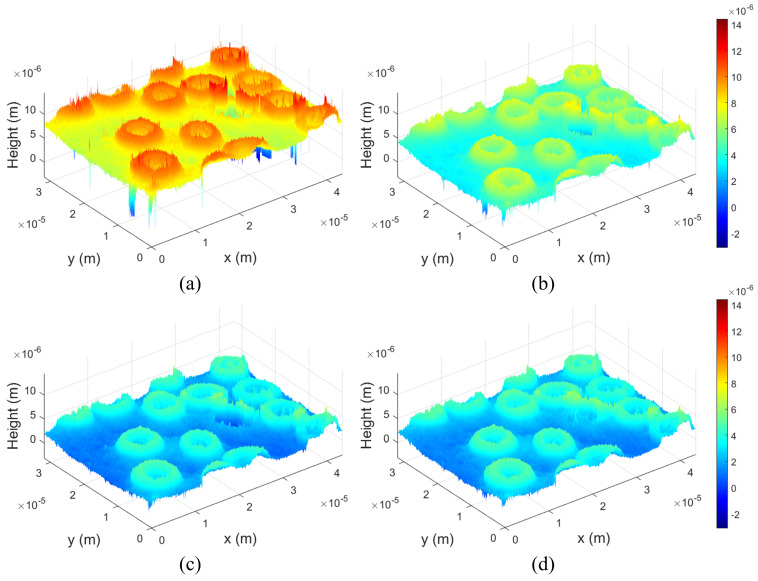
Reconstructed 3D profiles obtained by the proposed method. (**a**) $N_{R}$ = 1, (**b**) $N_{R}$ = 5, (**c**) $N_{R}$ = 10 and (**d**) $N_{R}$ = 20.

Figure 6 shows the reconstructed 3D profile obtained by the RaCoHS algorithm with different $N_{R}$. Figure 6a is the result of selecting only one windowed sideband, so it is not a filtering result by the RaCoHS algorithm, but is 3D profile obtained by the conventional image processing method. As shown in Figure 6a, a lot of random noise is also observed, and we can recognize that the error of phase unwrapping is caused by this random noise. On the other hand, we can recognize that the proposed method reduces random noise even when $N_{R}$ is 5 as shown in Figure 6b. Furthermore, the value of $N_{R}$ increases, the random noise decreases and the error of phase unwrapping of the background is resolved as shown in Figure 6c,d. This is because random noise appears at different locations in 3D profile due to windowed sidebands sampled at different locations in the Fourier domain. Moreover, the overall height of the background gradually approaches 0 as $N_{R}$ increases as shown in Figure 6. Therefore, as the number of samples increases, more accurate 3D information of the object can be obtained. We may not say that the optimal $N_{R}$ value is 20, and when the $N_{R}$ value is higher than 20, the result will converge to an accurate 3D profile. However, since the image processing time increases in proportion to the $N_{R}$ value, users can select an appropriate $N_{R}$ value according to needs. As shown in Figure 6, it can be seen that the slide glass is tilted. However, because the target of DHM is a micro-scale object, it is difficult to set up an accurate horizontal level, and accurate height information can be obtained by the relative height difference with the background. Therefore, the tilt of the slide glass does not have a significant effect on the height information of the target.

Since it is not possible to verify the exact effect of the proposed algorithm just by visually checking 3D profile, it is necessary to create an ideal 3D profile as a mathematical model and compare and analyze it. As mentioned in Section 3, we use uniform microspheres for numerical analysis. In addition, we use constant refractive indices of microspheres ($n_{o}$) and surrounding medium ($n_{s}$) with 1.5983 (refractive index of the polystyrene in 532 nm wavelength) and 1.49 [18], respectively.

**Figure 7:**
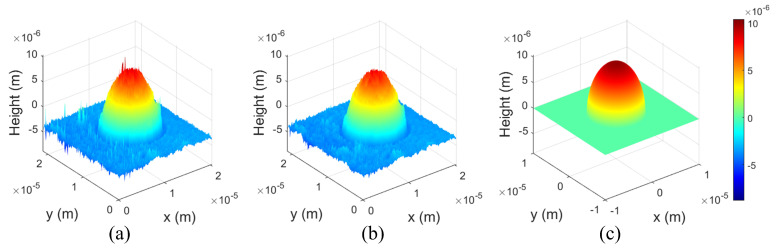
Reconstructed 3D profile using microsphere. (**a**) The conventional method, (**b**) the proposed method ($N_{R}$ = 20), and (**c**) the ideal comparison model of the microsphere in DHM.

Figure 7 shows reconstructed 3D profiles using a microsphere. Figure 7a,b show 3D profiles by the conventional method and the proposed method ($N_{R}$ = 20), respectively. In addition, Figure 7c shows the ideal comparison model of microsphere in DHM. In transmissive type DHM, 3D profile cannot classify the lower part and the upper part of the real object as shown in Figure 7c [18]. In this experiment, we select 10 different 3D profiles of the microsphere and compare them with the conventional method. We conducted a numerical analysis using metrics such as Structure similarity (SSIM) and Mean square error (MSE) in this paper [33,34].

Table 1 shows the result of the numerical analysis using SSIM and MSE. By checking the average value of each measurement result, we can verify that the proposed method has less noise than the conventional method and can create a 3D profile closer to the ideal comparison model.

**Figure 8:**
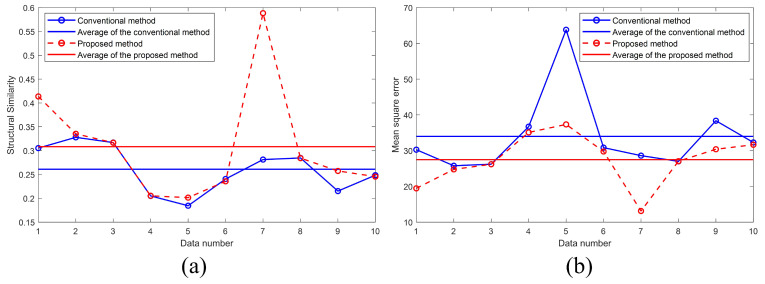
Result of the numerical analysis. (**a**) SSIM and (**b**) MSE.

Figure 8 shows the result of the numerical analysis. As shown in Figure 8a, the proposed method is closer to the ideal comparison model than the conventional method. Moreover, it can be recognized that the proposed method has less noise than the conventional method as shown in Figure 8b. Besides, in several samples (the sample number 2, 3, 4, 6, 8, and 10), the effect of the proposed method is not significantly different from that of the conventional method, and in other samples (the sample number 1, 5, 7, and 9), the effect of the proposed method shows a lower amount of noise and higher SSIM values compared to the conventional method as shown in Figure 8. This is because the amount of noise in 3D profile reconstructed by each recorded hologram is determined randomly. As a result, the proposed method shows better results in both metrics compared to the conventional methods in terms of average values (Horizontal lines in Figure 8a,b).

## 5. Conclusions

In this paper, we have proposed a novel DHM image processing method for randomly selecting the center of the high-frequency sideband in the Fourier domain to improve the accuracy of disease diagnosis using RBCs. In addition, we have compared 3D profile by the conventional method and 3D profile reconstructed by the proposed algorithm. As a result, we have verified that the proposed algorithm can reduce random noise while maintaining high-frequency information compared to conventional image processing methods. Besides, we have compared with representative filtering algorithms. We have verified that the proposed method shows a similar effect to the conventional filtering method while using only the windowed sideband including high-frequency information. Then, to confirm this effect, the ideal 3D profile of microspheres of uniform size has been modeled, compared, and analyzed. Therefore, the proposed method has a similar effect to the conventional filtering method of filtering while crushing high-frequency information, but has the advantage of maintaining high-frequency information. In addition, the proposed filtering algorithm not only provides shape information similar to the ideal comparison target compared to conventional methods, but also corrects depth information about the background of the object. Moreover, the proposed filtering algorithm has the advantage of being able to filter without loss of information using only information from recorded hologram images, unlike conventional digital holographic image processing methods that remove DC spectrum [35] or filter high-frequency amplitudes [15,17].

Of course, the proposed method has a disadvantage in that the image processing time increases in proportion to the $N_{R}$ value compared to the conventional methods. However, if a system capable of diagnosing a disease within a short time with just a drop of the tester’s blood is completed, the diagnosis of the presence or absence of a disease can be accelerated, which can increase human life expectancy. Therefore, it can be said that there is a need for research to increase the accuracy of diagnosis by filtering while maintaining high-frequency information as much as possible. For this reason, the proposed algorithm has many advantages over conventional image processing methods of digital holography, including DHM, and can be an important method in improving the accuracy of disease diagnosis. 

## Figures and Tables

**Table 1:** Numerical comparison by calculating SSIM and MSE.

	SSIM		MSE	
**Data Number**	**Conventional Method**	**RaCoHS**	**Conventional Method**	**RaCoHS**
1	0.3053	0.4137	30.27	19.46
2	0.3279	0.3353	25.78	24.76
3	0.3169	0.3166	26.22	26.20
4	0.2050	0.2053	36.71	35.09
5	0.1845	0.2014	63.80	37.33
6	0.2405	0.2356	30.84	29.76
7	0.2812	0.5883	28.60	13.12
8	0.2847	0.2843	27.00	27.05
9	0.2152	0.2574	38.37	30.39
10	0.2485	0.2455	32.29	31.65
Average	0.2610	0.3084	33.99	27.48

## Data Availability

Data are contained within the article.

## Abbreviations

The following abbreviations are used in this manuscript:

3D	Three-dimensional
DHM	Digital holographic microscopy
DC	Direct current
MSE	Mean square error
NA	Numerical aperture
RaCoHS	Randomly selects the center of the high-frequency sideband
RBCs	Red blood cells
SSIM	Structure similarity
